# Visceral Kaposi's Sarcoma Related to Human Herpesvirus-8 in Liver Transplant Recipient: Case Report and Literature Review

**DOI:** 10.1155/2012/137291

**Published:** 2012-12-19

**Authors:** H. Benhammane, G. Mentha, E. Tschanz, O. El Mesbahi, P. Y. Dietrich

**Affiliations:** ^1^Department of Medical Oncology, Hassan II University Hospital, Fez, Morocco; ^2^Department of Transplantation, University Hospitals of Geneva, 1205 Geneva, Switzerland; ^3^Department of Pathology, University Hospitals of Geneva, 1205 Geneva, Switzerland; ^4^Department of Medical Oncology, University Hospitals of Geneva, 1205 Geneva, Switzerland

## Abstract

*Background*. Kaposi's sarcoma (KS) in transplant recipients is about 400 to 500 times rate in the general population. It is strongly associated to Human herpesvirus-8 (HHV-8) infection which has been found in 95% of KS lesions. The optimal approach to managing posttransplantation KS is to reduce or discontinue immunosuppressive therapy but this strategy carries a risk of the acute rejection of the graft. Recently, the use of an mTOR inhibitor has added new opportunities for KS treatment and prevention. *Case Report*. We report a case of 24 years-old Turkish woman with visceral HHV-8-associated Kaposi's sarcoma after orthotopic liver transplantation. *Conclusion*. Posttransplantation KS is considered an experimental model of virus induced tumor suggesting the usefulness of HHV-8 screening in transplant recipient and donor. Therapeutic approaches are complex and require a multidisciplinary team.

## 1. Introduction

 Organ transplantation is associated with an increased risk of malignancies; Kaposi's sarcoma (KS) accounts for 5,7% of these malignancies [1]; it is more common in liver transplant recipients than in other solid organ transplantation; in a large Italian cohort on 417 liver transplant recipient, KS accounts for 14% of postliver transplant malignancies [2, 3]. This neoplasia is characterized by the predominance of skin and mucosal lesions; visceral extension is seen in only 10% of cases [2, 4, 5]. Human herpes virus-8 (HHV-8) is considered an essential, although not sufficient, etiologic agent for the development of KS; in the setting of organ transplantation the pathogenesis of this oncogenic virus remains not clearly understood [6]. The reduction or cessation of immunosuppressive therapy should be the first therapeutic maneuver of post-transplantation KS [1, 7]; recently, some reports demonstrate that the use of an mTOR inhibitor may be associated with a complete regression [8, 9]. Chemotherapy is an option for no responding patients. The role of antiviral therapy against HHV-8 is still controversial [6]. Herein, we report a case of visceral HHV-8-associated KS in 24 years-old woman after orthotopic liver transplantation (OLT) in order to underline the features and outcomes of this malignancy and to review the role of HHV-8. 

## 2. Case Report

**Figure 1:**
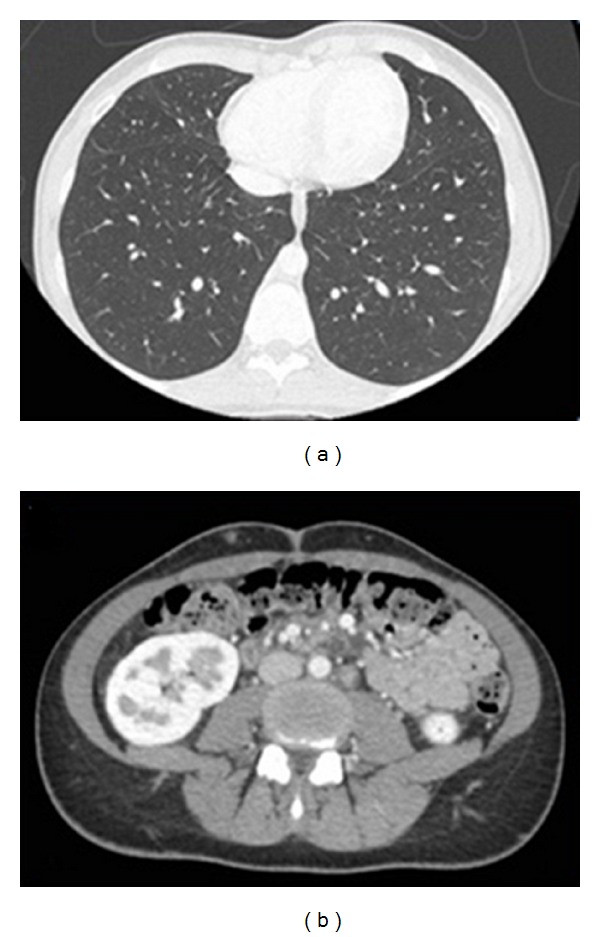
Computed tomography showing multiple pulmonary nodules (a) and retroperitoneal lymph nodes (b).

**Figure 2:**
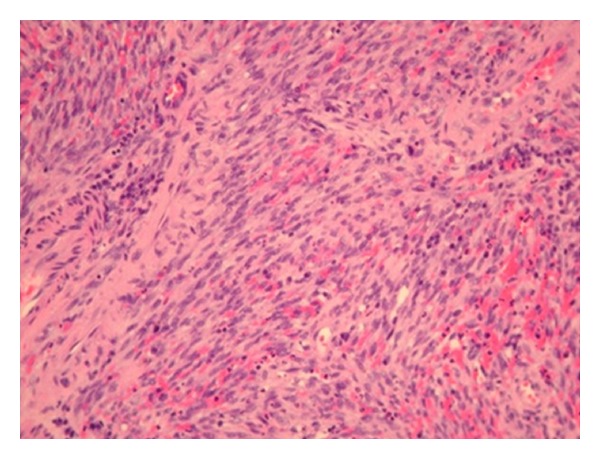
Microscopic findings of small bowel specimen showing atypical spindle cells with extravasated erythrocytes and capillaries (HES ×100).

**Figure 3:**
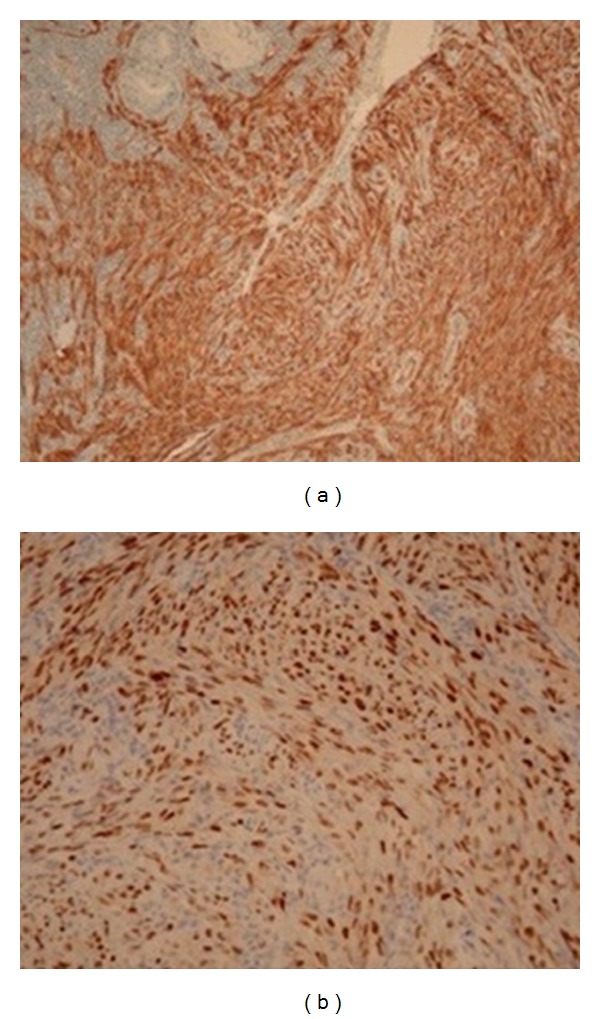
Immunohistochemical analysis showing the positivity for CD31 and CD34 (a) and the strong immunoreactivity for HHV-8 in the nuclei (b).

A 24 years-old Turkish woman underwent OLT in November 2008 for cirrhosis related to Von Gierke disease. Her immunosuppressive regimen consisted of tacrolimus and mycophenolate mofetil. Three years later, she was readmitted because of a cholestasis due to the stenosis of the biliobiliary anastomosis. Endoscopic Retrograde Cholangiopancreatography with placement of biliary stent was performed, but the procedure was complicated by pancreatitis and duodenal ulcer; therefore, a biliary repair surgery was decided. A computed tomography performed preoperatively allowed to fortuitous detection of several pulmonary nodules and multiple intraperitoneal lymph nodes (Figure 1). The patient underwent laparotomy which demonstrated the presence of multiple lymph nodes of variable size, associated with a 3 cm sized lesion of small bowel; principal bile duct was dilated secondary to a biliary nevrome. Segmental resection of small bowel with lymphadenectomy was performed; biliary stenosis was treated by hepaticojejunal anastomosis on Roux-en-Y. Histological analysis confirmed the diagnosis of kaposi's sarcoma of small bowel with lymph nodes extension (Figure 2); tumoral cells showed strong positivity in the nuclei for HHV-8, CD34 and CD31 (Figure 3). Antibody testing for HHV-8 was reactive on the serum bank collected on day 5 of OLT excluding a donor related infection. The serum HHV-8 viral load was 989 copies/Ml. other viral serology (HIV, hepatitis B and C virus, Epstein-Barr virus and cytomegalovirus) was negative. The immunosuppressive regimen was initially reduced, by stopping mycophenolate mofetil and halving the oral tacrolimus daily dose from 5 to 2 mg during 1 month then it was converted to everolimus at a daily dose of 2 mg. Currently, the patient remains well without sign of progression disease 9 months after surgery and with a good tolerance of treatment.

## 3. Discussion

Kaposi's sarcoma (KS) is about 400–500 times more common in transplant recipients than in general population [7], with an overall incidence of 0,4–6% in the United States and Western Europe [1, 10]. KS following organ transplantation and immunosuppressive therapy was first published in 1969 [11]. As reported in the Cincinnati Transplant Tumor Registry (CTTR), It accounts for 5 to 7% of all malignancies in transplant recipients when nonmelanoma skin cancer and in situ carcinoma of the uterine cervix are excluded [1]. 70% of posttransplant KS are diagnosed in the first 2 years after receiving transplantation and the most cases occur in individuals of Mediterranean, Jewish, Arabic, black, and Greek descent [6]. 

The incidence of KS is greater in liver transplant recipients (1,24%) than in heart (0,41%) or kidney (0,45%) transplantation [3, 4, 6] due to different types of immunosuppressive therapy. Clinical features are characterized by a predominance of cutaneous and mucosal lesions as multiple vascular nodules; an extension to viscera or lymph nodes is observed in only 10% of cases which 50% occur in liver transplant recipient [2, 4, 5]. Visceral disease has a substantial mortality ranging from 30–60% [1].

Although the pathogenesis of KS in transplant patients is poorly understood, a strong correlation with immunosuppressive status and the reactivation of HHV-8 was demonstrated. In 1994, HHV-8 has been isolated in all four types of KS (classic, endemic, organ transplant-associated, and AIDS-related KS) [6]. Over 95% of KS lesions, regardless of their clinical type, have been found to be infected with HHV-8. this oncogenic virus is an herpesviridae family strongly associated to KS and other conditions especially Castleman's disease and primary effusion lymphoma. In the endemic area for HHV-8, graft recipients who develop KS are generally seropositive before transplantation as is the case of our patient, but a transmission from donors to recipients has been documented [12]. The following explanations have been suggested: viral reactivation due to immunosuppressive therapy and chronic immunologic reaction between altered lymphoid cells and normal lymphocytes; in fact, HHV-8 infects both lymphatic and blood vascular endothelial cells and seems to target immunosuppressor tumor proteins (retinoblastoma and p53) causing the production of lymphangiogenic growth factors, (bFGF, Scatter factor, IL6, etc), endothelial cell proliferation, and tumorigenesis [12, 13]. Therefore post-transplantation KS represents actually an experimental model of virus induced tumor as a consequence of prolonged immunosuppression. 

Although, infection with HHV-8 is essential but not sufficient for the development of KS in transplant recipient, several cofactors have been identified including extent and duration of immunosuppression, reduced levels of neutralizing antibodies, poor cytotoxic T-cell response to HHV-8, and concomitant viral infection (HIV, hepatitis B virus, Epstein-Barr virus, cytomegalovirus, and herpes simplex virus) [12, 13]; additionally, when post-transplantation is more common in some ethnic background, several reports suggest a genetic predisposition related to some HLA phenotype [12]; however, because of the absence of specific studies on HLA phenotype in organ transplant recipient and donor, this hypothesis remains controversial.

No consensus on the optimal treatment for post-transplantation KS is available; therapeutic choice is often made based upon the experience and medical discipline; many treatment modalities have been used: surgical excision, radiation therapy, intralesional injection of chemotherapeutic agents, reduction of immunosuppressive therapy and systemic chemotherapy. Because the disease is a consequence of prolonged immunosuppression, the reduction or cessation of immunosuppressive therapy may be the most effective and reasonable approach [1, 6, 12], in the CTTR, a complete remission was observed in 42% of patients after various treatment; 38% of these remissions followed the reduction or cessation of immunosuppressive therapy; nonvisceral disease was associated to a higher remission rate than visceral disease (53% versus 27%) [1]. Similar results were reported by other authors [7, 12]. However, the management of immunosuppression is difficult particularly in KS following liver or heart transplantation [12] and requires a balance between the risk of death from neoplasia and the risk of graft rejection due to under immunosuppression [7]. 

Recently, some data suggest that switching to immunosuppressive therapy using a (Sirolimus or Everolimus) can induce the regression of iatrogenic KS through antiangiogenic activity related to impaired VEGF production and the limited proliferative response of endothelial cells to VEGF stimulation [8, 9]; additionally, L. A. Nichols showed a probable role of mTOR inhibitor in the prevention of KS in immunosuppressed patient by blocking HHV-8 production via the regulation of replication and transcription activator expression [14]. Given the complete regression reported in several published cases, we think that mTOR inhibitor should be included in the treatment of post transplantation KS. When the reduction or cessation of immunosuppressive therapy failed, conventional chemotherapy can be used. Pegylated liposomal doxorubicin is considered to be the first line therapy of choice leading to 70% of complete or major response approximately [15]. The other cytotoxic agents that seem to be effective in KS are vinblastine, bleomycin, taxane, etoposide, and gemcitabine. There are a few data on the role of antiviral therapy against HHV-8, but in combination with other treatments, they may allow to a satisfactory response as noted in some available observational reports [16].

## 4. Conclusion

The significant increase in the incidence of KS in transplant recipients shows the role of the immune system in the control of HHV-8 infection which is considered the principal, but not sufficient, etiologic agent for this neoplasia; there is evidence that screening transplant recipients and blood donors for HHV-8 infection may be beneficial. Further, studies are needed to identify immunologic, infectious and genetic cofactors possibly involved. The reduction of immunosuppression is a useful therapeutic approach of post-transplantation KS; the introduction of an mTOR inhibitor may permit the remission of the sarcoma; however, the total regression of the lesions should not be the goal because of the risk of graft rejection. In fact, therapeutic approaches are complex and require a skilled, experienced, multidisciplinary team.
